# Plasma Heme Oxygenase-1 Levels Distinguish Latent or Successfully Treated Human Tuberculosis from Active Disease

**DOI:** 10.1371/journal.pone.0062618

**Published:** 2013-05-06

**Authors:** Bruno B. Andrade, Nathella Pavan Kumar, Katrin D. Mayer-Barber, Daniel L. Barber, Rathinam Sridhar, Vaithilingam V. Banu Rekha, Mohideen S. Jawahar, Thomas B. Nutman, Alan Sher, Subash Babu

**Affiliations:** 1 Laboratory of Parasitic Diseases, National Institutes of Health, Bethesda, Maryland, United States of America; 2 National Institutes of Health, International Center for Excellence in Research, Chennai, India; 3 Government Stanley Medical Hospital, Chennai, India; 4 National Institute for Research in Tuberculosis, Chennai, India; 5 Science Applications International Corporation, National Cancer Institute, Frederick, Maryland, United States of America; University of Sassari, Italy

## Abstract

**Background:**

Tuberculosis (TB) is associated with oxidative stress and the induction of host anti-oxidants to counteract this response. Heme oxygenase-1 (HO-1) is a critical promoter of cytoprotection in diverse disease models including mycobacterial infection. Nevertheless, the pattern of expression of HO-1 in human tuberculosis has not been studied. Here, we examine expression of HO-1 in *M. tuberculosis*-exposed and -infected individuals and test its ability to distinguish active from latent and successfully treated TB cases. In addition, we assess correlations between plasma levels of HO-1 and cytokines closely associated with the immunopathogenesis of TB.

**Methods:**

Cross-sectional and longitudinal analyses of levels of HO-1, acute phase proteins and pro-inflammatory cytokines were performed in plasma samples from individuals with active pulmonary, extra-pulmonary or latent TB infection and healthy controls as part of a prospective cohort study in South India.

**Results:**

Systemic levels of HO-1 were dramatically increased in individuals with active pulmonary and extra-pulmonary tuberculosis and particularly those with bilateral lung lesions and elevated bacillary loads in sputum. HO-1 levels effectively discriminated active from latent tuberculosis with higher predictive values than either C-reactive protein or serum amyloid protein. Moreover, there was a marked reduction in HO-1 levels in active TB cases following anti-tuberculous therapy but not in those who failed treatment. Pulmonary TB patients displaying the highest concentrations of HO-1 in plasma exhibited significantly elevated plasma levels of interleukin (IL)-10, interferon (IFN)-γ and IL-17 and diminished levels of tumor necrosis factor (TNF)-α.

**Conclusion:**

These findings establish HO-1 levels as a potentially useful parameter for distinguishing active from latent or treated pulmonary tuberculosis, that is superior in this respect to the measurement of other acute inflammatory proteins.

## Introduction

The diagnosis of tuberculosis (TB) is a complex and lengthy process that involves the integration of clinical, radiological and microbiological parameters. The most widely used technique for confirming the presence of TB is the microscopic detection of acid-fast bacilli (AFB) in sputum, that has a sensitivity ranging between 34–80% [1]. Although sputum cultures (both solid and liquid) are more sensitive and accurate than sputum smears, they are time consuming and often difficult to implement in resource limited regions highly endemic for TB. Another major challenge in TB diagnosis is the ability to distinguish active from latent TB infection (LTBI) [2]. In this regard, the tuberculin skin test (TST) and interferon-gamma (IFN-γ) release assays, while useful for identifying individuals exposed to *Mycobacterium tuberculosis*, in themselves cannot be used for accurately establishing a diagnosis of active TB [3]; typically the diagnosis additionally requires identification and/or culture of bacilli in biological fluids or tissue biopsies. Importantly, approximately 15% of individuals with clinical symptoms of TB have no microbiologic confirmation [4], and, in such cases, the empirical response to anti-tuberculous therapy (ATT) is frequently used for diagnostic confirmation. Thus, the identification of host biomarkers for rapid and accurate diagnosis of active TB and for assessing successful responses to therapy remains a critical need in the clinical management of active TB.

Studies in patients with active TB have demonstrated decreased systemic concentrations of antioxidants and enhanced spontaneous generation of free radicals compared to individuals without TB [5] reflecting the excessive oxidative stress associated with this disease. Heme oxygenase-1 (HO-1) is a major anti-oxidant highly expressed in lung tissue and is a key stress-response enzyme that degrades heme molecules thereby releasing free iron, carbon monoxide (CO) and biliverdin [6]. Recently, HO-1 has been established as a critical determinant of cytoprotection because of the anti-inflammatory and anti-oxidant properties mediated by CO and biliverdin [7]. Increased expression of HO-1 has been observed in the plasma of individuals with a variety of pulmonary pathologies, including acute respiratory distress syndrome, chronic obstructive pulmonary disease and asthma (reviewed in [8]). Elevations of HO-1 have also been observed in several other conditions not specifically localized to the lung such as malaria [9], leishmaniasis [10] and sepsis [11]. In these disorders, plasma levels of HO-1 frequently associate with the degree of disease severity.

Experimental studies have demonstrated that *M. tuberculosis* infection triggers expression of HO-1 in mouse macrophages in vitro and lung tissue in situ [12], [13]. In addition, it has been shown that HO-1 promotes granuloma development and host resistance to *Mycobacterium avium* in mice [14]. Nevertheless, the role of HO-1 in the pathogenesis of active TB in humans has not been systematically studied. In the present report, we have examined the expression of HO-1 in a cohort of *M. tuberculosis*-exposed and -infected individuals from South India which has a high TB burden [15] and where recent studies have evaluated the accuracy of microbiological tools for TB diagnosis [16]. Our findings demonstrate that plasma HO-1 levels can be used to distinguish active TB from both latent infection and healthy uninfected individuals with remarkably high accuracy. Moreover, HO-1 levels correlate with bacillary burden in sputum and accurately reflect treatment efficacy in patients receiving chemotherapy.

## Methods

### Ethics Statement

Written informed consent was obtained from all participants or their legally responsible guardians, and all clinical investigations were conducted according to the principles expressed in the Declaration of Helsinki. The clinical protocols from which the plasma samples were used in the present study were approved by the Institutional Review Board of the National Institute of Research in Tuberculosis (NCT01154959 and NCT00342017).

### Study Design and Participants

Plasma samples were collected from 97 patients with active pulmonary TB (PTB), 35 patients with extra-pulmonary TB (EPTB), 39 individuals with LTBI and 40 healthy donors recruited in Chennai, India, as part of a TB cohort study. Active TB and LTBI cases were recruited at the Government Stanley Medical Hospital, and at TB clinics supported by the National Institute for Research in Tuberculosis, Chennai, India.

The diagnosis of PTB was based on sputum smear and culture positivity. EPTB was diagnosed on the basis of AFB staining and/or culture positivity of fine-needle aspiration biopsies of lymph nodes or pleural effusions. At the time of enrollment, all active TB cases had no record of prior TB disease or ATT.

LTBI diagnosis was based on TST and Quantiferon TB-Gold ELISA positivity [17], absence of chest radiograph abnormalities or pulmonary symptoms and negative sputum smears or cultures. A positive TST result was defined as an induration at the site of tuberculin inoculation of at least 12 mm in diameter to minimize false positivity due to exposure to environmental mycobacteria [18]. Healthy donors were asymptomatic with normal chest X-rays, negative TST (indurations <5 mm in diameter) and Quantiferon as well as negative sputum smear or culture results. All participants were BCG vaccinated and were HIV negative. The study groups were similar with regard to age and gender and the baseline characteristics of the study participants are shown in Table 1.

**Table 1:** Demographic characteristics of the study participants.

Parameter	Healthy donors	Latent TB infection	Extra pulmonary TB	Pulmonary TB	P value
N	40	39	35	97	
Median Age (IQR) -y	29 (21–59)	25 (21–49)	33 (18–65)	40 (19–70)	NS
Male (%)	25 (62.5)	23 (59)	16 (45.7)	67 (69.1)	NS
Positive Quantiferon test (%)*	0 (0)	39 (100)	25 (71.4)	65 (67.0)	NS
Extra-pulmonary infection site (%)†					0.01
*Neck*	N/A	N/A	20 (58)	N/A	
*Chest/mediastinum*	N/A	N/A	11 (31)	N/A	
*Breast*	N/A	N/A	4 (11)	N/A	

NOTE: Age distribution between the groups was compared using Kruskal-Wallis test. Percentage of male individuals and positive Quantiferon tests were analyzed using chi-square and Fisher’s exact tests respectively. IQR, interquartile range; NS, non significant; TB, tuberculosis.

* Healthy donors and latent TB infection cases were not included in the statistical analysis for Quantiferon positivity, as the test status was part of the criteria to categorize these groups.

† Percentage of individuals presenting with different sites of infection with the extra-pulmonary tuberculosis group were compared using chi-square test.

ATT was given to all patients with active PTB and EPTB using the directly observed treatment, short course (DOTS) strategy following the World Health Organization (WHO) guidelines [19]. At 6 months following ATT initiation, new fresh plasma samples were obtained. Those PTB patients who maintained positive sputum cultures at 6 months of ATT were considered to be treatment failures following the current WHO definition [19].

### Measurement of Plasma HO-1, Cytokine and Acute Phase Protein Levels

HO-1 (Assay Designs, Ann Arbor, MI), interleukin (IL)-10, tumor necrosis factor (TNF)-α, IFN-γ and IL-17 (R&D Systems, Minneapolis, MN) in plasma were measured by ELISA. Levels of C-reactive protein (CRP) and Serum Amyloid Protein-A (SAA) were determined using the Bioplex (Bio-Rad, Hercules, CA) multiplex ELISA system according to the manufacturer's instructions.

### Data Analysis

The median values with interquartile ranges (IQR) were used as measures of central tendency. HO-1 levels were compared between the study clinical groups using the Kruskal-Wallis test with Dunn’s multiple comparisons or linear trend post-test. The Mann-Whitney test was used to compare HO-1 concentrations between the individuals with pulmonary TB with unilateral or bilateral lung lesions. This test was also used to compare HO-1 levels between patients with positive AFB staining in the sputum smears and those with negative smears. The Wilcoxon matched pairs test was used to evaluate the significance of the effect of ATT on HO-1 values. The chi-square or Fisher’s exact tests were used to compare nominal values or data displayed as percentage between the study groups. Receiver Operator Characteristics (ROC) curves were designed to test the power of each candidate marker to distinguish LTBI from active TB. Univariate linear regression analyzes were performed to assess the odds ratios (OR) of the associations between HO-1 and active TB or pulmonary TB. Correlations between cytokines, acute phase proteins and HO-1 were evaluated using Spearman rank sign test. Linear curve fit was used to illustrate the trends of the correlations in the graphs. The statistical analyses were performed using and STATA 10 (StataCrop LP, College Station, TX) and graphs were plotted using Graphpad Prism 5.0 (GraphPad Software, San Diego, CA).

## Results

### Plasma HO-1 is Elevated in Patients with Active Tuberculosis

**Figure 1:**
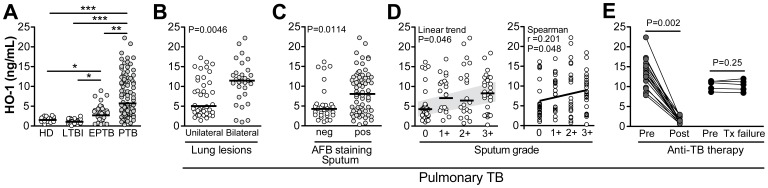
HO-1 levels associate with disease severity and sputum positivity in tuberculosis. (**A**) HO-1 levels were evaluated in plasma samples from 97 individuals with active pulmonary tuberculosis (PTB), 35 with active extra pulmonary tuberculosis (EPTB), 39 with latent *M. tuberculosis* infection (LTBI) and 40 healthy donors (HD). The samples were collected prior to initiation of anti-tuberculosis treatment (ATT). Kruskal-Wallis test and Dunn’s multiple comparisons were used to evaluate statistical differences between the groups. (**B**) HO-1 levels were compared between 40 PTB patients with a diagnosis of unilateral lung lesions and 37 patients with bilateral lesions identified by chest radiography. (**C**) PTB cases were classified according to the presence or absence of the acid fast staining bacilli (AFB) in sputum samples. In (**B**) and (**C**), the Mann Whitney test was used for statistical comparisons. (**D**) PTB cases were stratified according to the quantitative bacillary sputum grade determined by AFB staining. Data was analyzed using the Kruskal-Wallis test with linear trend post-test (left panel) or by Spearman correlation (right panel). In (**A**), (**B**), (**C**) and (**D**), bars represent median values. (**E**) Comparison of HO-1 levels pre and post ATT in a subset of patients with PTB for which plasma samples were available (n = 33). Data from 5 patients that failed treatment are also shown. The Wilcoxon matched pairs test was used to evaluate the significance of the effect of ATT on HO-1 values. Tx, treatment. *P<0.05; **P<0.01; ***P<0.001.

Individuals with active pulmonary or extra-pulmonary TB displayed significantly higher systemic levels of HO-1 (medians [IQR]: 5.8 [3.2–11.6] ng/mL and 3.45 [2.0–4.5] ng/mL, respectively; P<0.01) than either individuals with latent TB or healthy controls, who expressed only marginal concentrations of the enzyme (medians [IQR]: 1.3 [0.78–1.5] ng/mL and 1.4 [1.0–1.9] ng/mL, respectively; P = 0.49; Figure 1A). Among those with active pulmonary TB, individuals with bilateral lung lesions had higher systemic levels of HO-1 compared to patients with unilateral lesions as identified by chest radiography (P = 0.005; Figure 1B), indicating a possible relationship between HO-1 and the anatomical extent of the disease. Moreover, the majority (23/40) of patients with unilateral lung lesions had negative AFB staining in sputum smears whereas most (30/37) with bilateral lesions had positive smears. Thus, AFB sputum positivity was associated with bilateral lung lesions in this study population (Fisher’s exact test P<0.001; OR: 3.17, 95% confidence interval [CI]: 1.13–8.91). The systemic levels of HO-1 were higher in individuals with positive AFB staining in the sputum than in those with negative smears (P = 0.01; Figure 1C). Interestingly, HO-1 concentrations tended to be more elevated in individuals with higher sputum bacillary grade (Linear trend analysis P = 0.046; Spearman correlation r = 0.2, P = 0.048; Figure 1D). Importantly, the elevated plasma concentrations of HO-1 in pulmonary TB patients returned to background levels (i.e., not significantly different from healthy controls) following successful anti-tuberculous chemotherapy (n = 33) (P<0.001; Figure 1E) but were not significantly altered in the small subset of patients (n = 5) who failed treatment as defined by positive sputum cultures at the end of the course of drug therapy.

**Figure 2:**
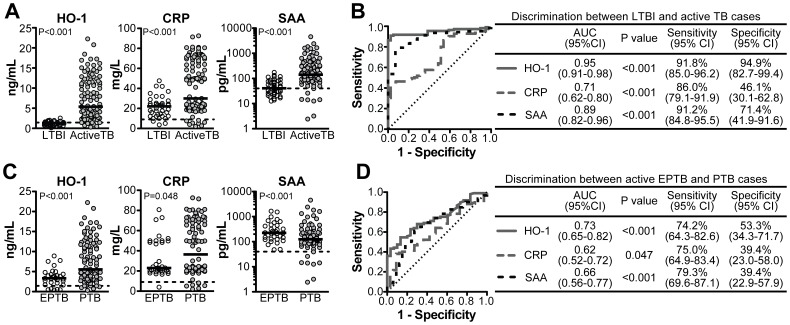
HO-1 as a candidate marker of active pulmonary tuberculosis. Levels of HO-1, C-reactive protein (CRP) and Serum Amyloid Protein-A (SAA) were compared between individuals with LTBI and active TB (**A**) or between patients with active EPTB and those with PTB **(C)** using the Mann Whitney test. The power of each of the three different biomarkers to discriminate the above clinical outcomes as analyzed by ROC curves is illustrated in (**B**) and (**D**). Symbols represent individual patients and bars represent median values. In (**A**) and (**C**), dotted lines represent median values from healthy donors. AUC, area under the curve; 95%CI, 95% confidence interval.

We next estimated the ability of HO-1 to discriminate between active TB and latent TB infection. Concurrently, we also compared the discriminatory power of HO-1 with two other markers of inflammation, CRP and SAA, previously described to be elevated during active TB [20]. Increased levels of HO-1, CRP and SAA were detected in plasma from patients with active TB (with PTB and/or EPTB) compared to individuals with LTBI (P<0.001; Figure 2A). Among these three candidate markers for active TB, HO-1 had the highest discriminatory power (Figure 2B) with a 23.5% higher specificity in distinguishing active from LTBI compared to SAA (94.9% vs. 71.4%, respectively) and 48.8% higher specificity compared to CRP (94.9% vs. 46.1%, respectively; Figure 2B). Moreover, the positive predictive value (PPV) in the context of TB for HO-1 was 98.9% (95% CI: 94.0–100%) and the negative predictive value (NPV) was 80% (95% CI: 61.5–81.9%). In addition, we compared the power of HO-1, CRP and SAA to discriminate between patients with PTB from those with EPTB. Systemic levels of HO-1 and CRP were significantly higher in PTB cases, while SAA concentrations were slightly but consistently reduced (Figure 2C). Further analysis revealed that HO-1 was the most powerful parameter for discriminating pulmonary from EPTB although with a reduced accuracy compared to that seen in the discrimination of active from latent infection (Figure 2D; PPV: 79.3%, 95% CI: 68.6–87.1%; NPV: 36.4%, 95% CI: 22.4–52.2%).

### Pulmonary TB Patients with Higher HO-1 Levels Display a Unique Cytokine Expression Profile

**Figure 3:**
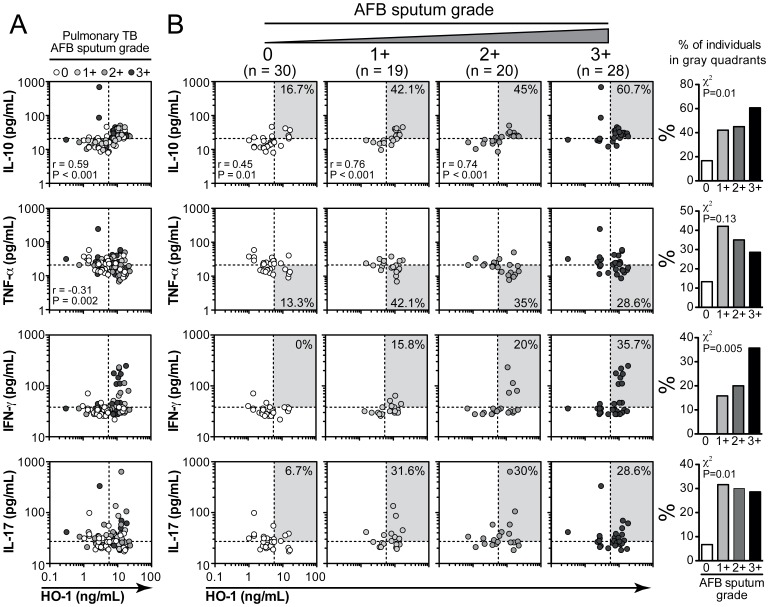
Associations between HO-1 and cytokine levels in active pulmonary tuberculosis. (**A**) Correlations between plasma HO-1 levels and systemic concentrations of IL-10, TNF-α, IFN-γ and IL-17 in individuals with active pulmonary tuberculosis (n = 97). Dotted lines on the X-axis represent the median value of HO-1 within the group of patients with pulmonary disease, while dotted lines on each Y-axis indicate median values for each cytokine. In (**B**), the correlations were stratified according to the bacillary load in sputum smears. Gray areas designate the quadrants that include the individuals simultaneously displaying values of HO-1 and cytokines above the medians or in the case of TNF-α below the median. Percentage of individuals within in the gray areas was compared between the groups with different bacillary sputum grades using a chi-square analysis and correlations evaluated using Spearman test.

We next tested if plasma HO-1 levels are associated with a specific pattern of systemic cytokine expression within the study population. We found that, in individuals with active PTB, levels of HO-1 were positively correlated with IL-10 levels (r = 0.59, P<0.001; Figure 3A) and negatively correlated with TNF-α levels (r = –0.31, P = 0.002; Figure 3A) in plasma. There were no significant associations between HO-1 and IFN-γ (r = 0.30, P = 0.05) or HO-1 and IL-17 levels (r = –0.02, P = 0.83) (Figure 3A). The strength of relationship between HO-1 and IL-10 was increased in those patients with higher bacillary burdens (Figure 3B). In addition, a significant percentage of individuals presenting with high values of HO-1 and low concentrations of TNF-α also exhibited positive sputum AFB smears (34.3% of the patients with positive smears vs. 13.3% of those with negative screening; Fisher’s exact test P = 0.2; OR: 3.9, 95% CI: 1.2–12.5; Figure 3B). Interestingly, the patients expressing the highest levels of IFN-γ or IL-17 in the plasma also expressed markedly elevated concentrations of HO-1 (Figure 3B). In addition, a significant percentage of individuals with simultaneously high levels of HO-1 and IFN-γ or IL-17 also exhibited a higher sputum AFB smear grade (chi square P = 0.005 and P = 0.01, respectively; Figure 3B).

## Discussion

Early and accurate diagnosis of active TB is an important first step in both patient treatment and the prevention of transmission, but the diagnostic tests currently available for this purpose have important shortcomings, particularly so in the settings of extra-pulmonary, sputum-smear negative and pediatric tuberculosis. For this reason the development of accurate host biomarkers that identify those individuals with active TB distinct from latent infection and can accurately monitor the response to chemotherapy has been designated an important research priority in TB research [21]. The present study reveals plasma HO-1 as a potential biomarker that appears to adequately fulfill these criteria. By analyzing a population in South India where TB is highly endemic, we have shown for the first time that HO-1 levels are able to identify individuals with active TB with remarkably high sensitivity, specificity and predictive values. The overall performance of HO-1 in discriminating between active and latent TB was significantly greater than either CRP and SAA, two highly sensitive markers of inflammation associated with TB [20]. Other soluble activation markers, such as intercellular adhesion molecule (ICAM)-1 [22], IL-2 receptor [23], neopterin [24], [25], beta 2-microglobulin [24] and IP-10 in blood [26] and urine [27], have also been shown to increase during infection and to decrease with treatment (reviewed in [21]), but none appear to display the predictive power of HO-1 demonstrated here. The explanation for this superior diagnostic performance is not clear but may relate to HO-1 being more reflective of excessive oxidative tissue damage, as suggested previously in a study of critically ill patients [11]. It is also possible that HO-1 preferentially detects pulmonary inflammation and does so more accurately than either CRP or SAA. The utility of HO-1 as a marker of active TB (either pulmonary or extrapulmonary) may be particularly useful in children where diagnosis often presents a challenge because of lack of access to sputum and the atypical clinical manifestations of pediatric TB, but this needs to be investigated.

Previous studies in *M. tuberculosis*-infected mice have shown that HO-1-derived carbon monoxide activates genes associated with the *M. tuberculosis* dormancy regulon [12], [13], suggesting that HO-1 is a trigger of latency. Our results demonstrating that HO-1 levels are higher in patients with elevated bacillary sputum burdens at first glance appear to be in conflict with this hypothesis. Although this discrepancy may reflect distinct functions for HO-1 in mouse versus humans, a more likely explanation is that it stems from a dissociation between the induction and activity of the enzyme during infection in vivo. In this regard, a variety of other mediators thought to contribute to the maintenance of latency in mice such as IFN-γ, TNF-α and nitric oxide [28], in common with HO-1, are also highly expressed in patients with active TB [29], [30] for reasons that are not entirely clear.

HO-1 is an intracellular enzyme expressed in many cell types and tissues. We hypothesize that in pulmonary TB patients, the increased HO-1 observed in plasma derives from injured tissues, a concept that would explain the strong association of HO-1 with both bacterial burden and disease severity in the lung. In addition to bacillary load and clinical outcome, HO-1 levels were also found to correlate positively with plasma IL-10 levels and negatively with TNF-α levels. This observation is consistent with previous studies in which HO-1 was shown to mediate the inhibitory effects of IL-10 on the expression TNF-α and other pro-inflammatory cytokines [31] and is also consistent with the known immunosuppressive effects of IL-10 on *M. tuberculosis* infection in vivo [32].

A major prerequisite of a reliable biomarker is the ability to reflect both disease activity and response to therapy. An important finding in the present study is that HO-1 levels mirror the success of chemotherapy when measured at the termination of ATT. This observation raises the interesting possibility that measurement of HO-1 at earlier time points might facilitate the identification of patients who respond to drug treatment more quickly and for whom shortened courses of therapy might be considered. Studies have been initiated to test this hypothesis and to determine whether HO-1 can also be used to predict relapse risk or treatment failure.

If the striking correlations between HO-1 and TB infection observed here can be validated in other populations and epidemiological settings, then a simple diagnostic test involving either an immunoassay to detect HO-1 or measurement of its enzymatic activity could be easily developed and leveraged in multiple ways for the clinical management of TB. Because HO-1 expression is known to be increased in patients with other lung pathologies, such a test may need to be optimized by combination with a secondary assay to provide an accurate read-out of lung disease in the specific context of *M. tuberculosis* infection.
